# Assessing Photosensitizer Targeting Using Meso-Tetra(Carboxyphenyl) Porphyrin

**DOI:** 10.3390/molecules23040892

**Published:** 2018-04-12

**Authors:** Upendra Chitgupi, Jonathan F. Lovell, Venugopal Rajendiran

**Affiliations:** 1 Department of Biomedical Engineering, University at Buffalo, State University of New York, Buffalo, New York, NY 14260, USA; upendrac@buffalo.edu; 2 Department of Chemistry, School of Basic and Applied Sciences, Central University of Tamil Nadu, Thiruvarur 610005, India

**Keywords:** porphyrin, photosensitizer, mTCPP, photodynamic therapy, PDT

## Abstract

Mesotetra(4-carboxyphenyl)porphyrin (mTCPP) is a commercially available small molecule fluorophore and photosensitizer with four free carboxylic acid groups. mTCPP can readily be conjugated with amines for facile attachment of functional groups. In this work, we synthesized and assessed tetravalent, lysine-conjugated mTCPP, for its potential applications in targeted imaging and photodynamic therapy. Fmoc-protected d-lysine or l-lysine was conjugated to mTCPP via amide coupling with the epsilon amine group of lysine, followed by Fmoc deprotection. The resulting compounds did not dissolve well in aqueous solvent, but could be solubilized with the assistance of surfactants, including cholic acid. The l-amino acid transporter (LAT1) can uptake diverse neutral l-amino acids. In vitro studies with U87 cells revealed a non-specific uptake of the hydrophobic Fmoc-protected lysine-conjugated mTCPP precursors, but not d- or l-lysine mTCPP. Likewise, only the Fmoc-protected compounds induced substantial phototoxicty in cells following incubation and irradiation with blue light. These experimental results do not provide evidence to suggest that lysine-mTCPP is able to specifically target cancer cells. However, they do highlight mTCPP as a convenient and accessible framework for assessing molecular targeting of photosensitizers.

## 1. Introduction

Photodynamic therapy (PDT) has been demonstrated as a minimally invasive ablative modality for cancer treatment [1,2]. Merits associated with PDT include low systemic toxicity, the ability to treat only light-targeted areas, and the possibility of undergoing repeated treatments without the development of resistance [3]. PDT requires several components to come together for activity: (i) a photosensitizer (PS) with a capacity to generate reactive oxygen species; (ii) light irradiation of the appropriate wavelength; and (iii) availability of oxygen in the target region [4]. Although many photosensitizers have been described, porphyrin-based ones have been the most commonly used in the clinic [5,6]. Hematoporphyrin derivative (Photofrin), an oligomeric porphyrin mixture, was the first photosensitizer used in modern clinical practice of PDT for anti-tumor treatment, and has been used extensively [7,8,9,10]. Although Photofrin is clinically approved for numerous solid tumors, it has some limitations such as prolonged skin sunlight sensitivity [11,12]. Since porphyrins typically generate fluorescence in the red or near infrared, there has been interest in using these for fluorescence imaging and theranostic applications [13,14]. Fluorescence imaging with PDT opens up intriguing possibilities, including image-guided therapies and feedback-driven light dosimetry [15].

There is ongoing research from chemical and biomedical perspectives to develop new photosensitizers with improved properties such as higher potency and reduced sunlight sensitivity. One approach to this end involves the targeting of photosensitizers to receptors found on tumors cells [16,17]. In order to exploit the binding affinity of these receptors with ligands, PDT agents coupled with ligands or encapsulated within functionalized, targeted particles have been developed [18,19]. Porphyrin- and chlorin-based PDT agents have been explored for active targeting by conjugating a wide range of targeting moieties, including antibodies, folic acid, sugars, and peptides [20,21,22,23,24,25,26,27,28,29].

Photosensitizer coupling approaches often make use of photosensitizers with single reactive functional groups for synthetic accuracy and purity. Unfortunately, there are few photosensitizers with single reactive functional groups available at low cost in bulk. Mono-l-aspartyl chlorin e6 (Npe6) is a tetracarboxylic photosensitizer that has been used in human PDT clinical trials. Meso-tetra(4-carboxyphenyl)porphyrin (mTCPP) is a low cost, commercially available, water soluble porphyrin derivative with four free carboxylic acid groups that are available for conjugation. As such, functionalization immediately provides multivalency, which has been demonstrated to improve targeting specificity [30]. mTCPP has been used in numerous applications including porphyrin metal organic frameworks [31], mesoporous silica [32], polymeric injectable theranostic probes [33,34,35], and polymeric implantable biosensors [36,37,38]. A structurally similar meso-tetra(4-hydoxylphenyl) porphyrin was used for functionalization and attachment of gadolinium chelates to produce a theranostic agent [39]. In this work, we make use of mTCPP for covalent conjugation of functional moieties for the purpose of photosensitizer targeting. 

Some tumor cell overexpress the membrane protein l-type amino acid transporter LAT1 (Na^+^ independent antiport transporter) [40,41]. Certain natural and artificial amino acids, anticancer drug melphalan, and BCH have been reported in the literature to have been effectively transported by the LAT1 system [42,43,44,45,46]. In the present work, we assess the targeting ability of mTCPP-lysine complexes to human glioblastoma (U87) cells that have been reported to express LAT1 expressed on their surface [47,48,49]. Our approach was to conjugate l-lysine amino acids via their epsilon amine group to the periphery of mTCPP. This design was speculated to then mimic the amino and carboxy groups connected to a hydrophobic core (the porphyrin) in a manner that might be recognized by LAT1 overexpressed on the surface of the cells. 

## 2. Results and Discussion

### 2.1. Synthesis and Formulation of Lysine-Conjugated Porphyrin 

**Figure 1:**
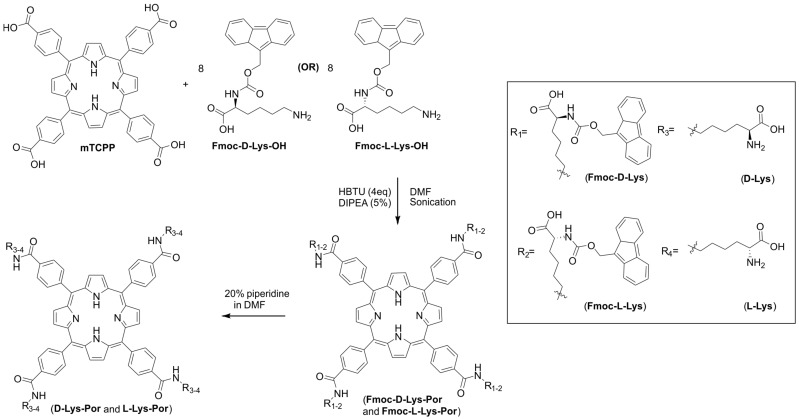
Synthesis of d-Lys-Por, l-Lys-Por, Fmoc-d-Lys-Por, and Fmoc-l-Lys-Por.

d-lysine or l-lysine was conjugated to mesotetra(4-carboxyphenyl) porphyrin (mTCPP) as shown in Figure 1. The procedure was a slightly modified synthetic procedure from our prior work which coupled amine-modified polyethylene glycol to mTCPP [35]. Fmoc-protected d- or l-lysine amino acids were first condensed with mTCPP in the presence of diisopropylethylamine (DIPEA) and HBTU in dimethylformamide (DMF). Deprotection of Fmoc groups was carried out by using 20% piperidine in DMF, and moderate yield was obtained (38–44%). Fmoc-d-Lys-Por and Fmoc-l-Lys-Por were characterized by visible and fluorescence emission spectroscopy, ^1^H-NMR (Figure S1), ^13^C-NMR (Figure S2), and high resolution mass spectrometry (Figure S3). Since the deprotected D-Lys-Por and l-Lys-Por were not completely soluble, they were characterized by UV-Visible and emission spectral methods in the presence of surfactants as indicated below. Although the NMR peaks were not visible for d-Lys-Por and l-Lys-Por even at a pH of 10, ^1^H-NMR spectrum showed the peaks when measured at higher temperature (80 °C) (Figure S4). Further, the purity of d-Lys-Por and l-Lys-Por were confirmed by HRMS spectral analysis (Figure S5).

**Figure 2:**
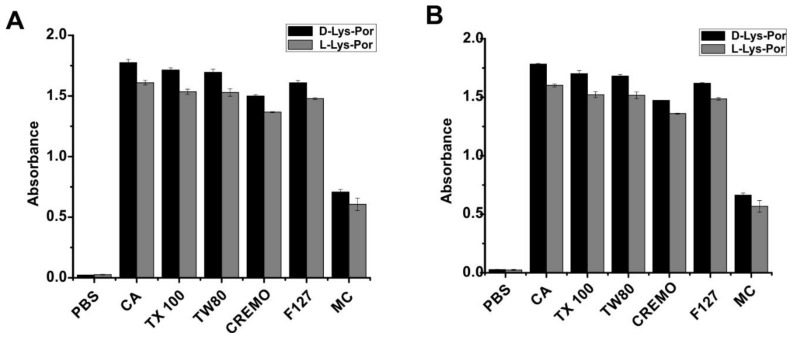
Effect of detergent on solubility of lysine-mTCPP complexes. Absorbance of d-Lys-Por and l-Lys-Por (5 μM) in 1% *w*/*v* solution of surfactants after (**A**) 1 h and (**B**) 12 h incubation. Pluronic F127 (F127), Cremophor EL (CREM), Tween 80 (TW80), sodium cholic acid (CA), methyl cellulose (MC), and Triton X-100 (TX 100) detergents were considered for the solubility studies of lysine complexes. Mean +/− std. dev. for *n* = 3.

The deprotected d-Lys-Por and l-Lys-Por compounds could partially dissolve in water but aggregated in a short span of time. However, both the compounds were found to be soluble in basic buffers of pH 10 and above. To avoid aggregation, various surfactants in phosphate buffered saline (PBS) pH 7.4, including Pluronic F127 (F127), Cremophor EL (CREM), Tween 80 (TW80), sodium cholic acid (CA), methyl cellulose (MC), and Triton X-100 (TX 100), were used to stabilize the compounds in aqueous medium. Optical absorbance was measured by dissolving 5 µM of d-Lys-Por and l-Lys-Por in 1% (*w*/*v*) surfactant in PBS. d-Lys-Por and l-Lys-Por dissolved in 1% CA exhibited slightly higher optical absorbance compared to other surfactants (Figure 2A). d-Lys-Por and l-Lys-Por dissolved in CA were centrifuged at 2000× *g* for 5 min, and no aggregates were seen, indicating that the samples were completely soluble. Absorbance of the samples in surfactants was measured 12 h later, and they exhibited similar optical absorbance as the initial time point (Figure 2B). Hence, the surfactants rapidly solubilized and stabilized the various lysine-mTCPP complexes. Fmoc-d-Lys-Por and Fmoc-l-Lys-Por were also found to be stable in CA. Therefore, unless otherwise noted, lysine-mTCPP stock solutions were prepared in PBS with 1% CA for subsequent studies.

### 2.2. Photophysical Studies

**Figure 3:**
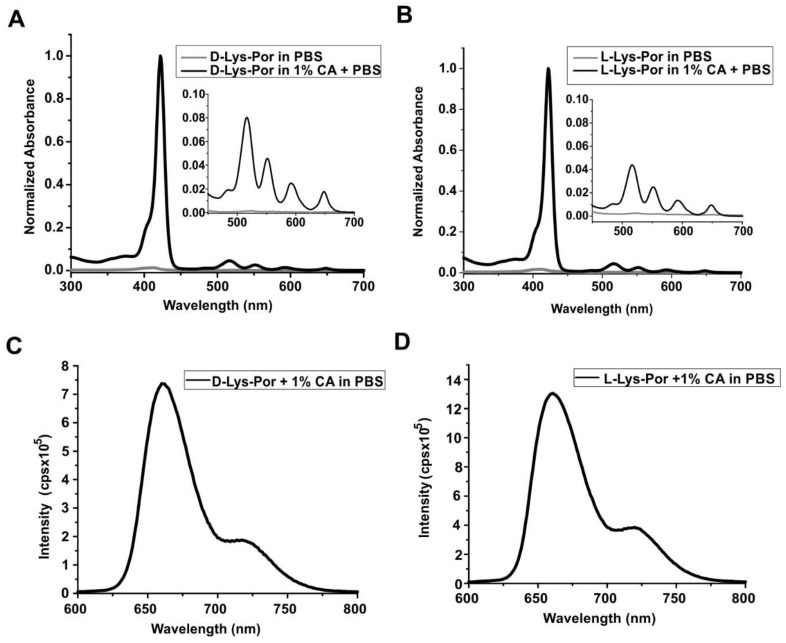
Spectroscopic characterization of lysine-mTCPP. UV-Visible spectra of (**A**) d-Lys-Por (**B**) l-Lys-Por in PBS and PBS with 1% CA. Emission spectra of (**C**) d-Lys-Por and (**D**) l-Lys-Por respectively in PBS with 1% CA.

In PBS and in the absence of CA, d-Lys-Por and l-Lys-Por did not show absorbance, due to poor solubility. However, when dissolved in PBS with 1% CA, both d-Lys-Por and l-Lys-Por (5 µM) displayed absorption spectra with a prominent Soret band and minor Q-bands apparent. d-Lys-Por and l-Lys-Por had a major Soret band around 421 nm and 427 nm, respectively, as shown in Figure 3. The Q-bands observed for d-Lys-Por and l-Lys-Por were at 521, 556, 591, and 649 nm (inset, Figure 3A) and 521, 557, 596, and 652 nm (inset, Figure 3B), respectively. The increase in intensity of the Soret and the Q-bands in the presence of CA can be attributed to increased solubility. There was no further increase in intensity with the increase in CA beyond 1%, indicating that the samples were completely solubilized with 1% CA in PBS. Both d-Lys-Por and l-Lys-Por showed a fluorescence emission peak around 660 nm (Figure 3C,D) when excited at 421 nm and 427 nm, respectively. 

**Figure 4:**
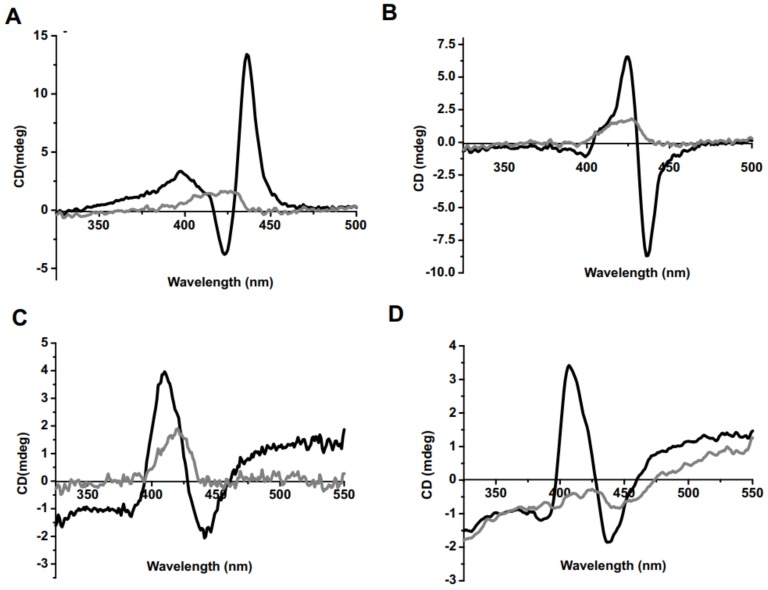
Circular dichroism (CD) spectra of (**A**) Fmoc-d-Lys-Por (10 µM) (**B**) Fmoc-l-Lys-Por (10 µM) in PBS with 0.2% DMSO in the presence of 0.02% CA (shown in black) and 0.5% CA (shown in gray) (**C**) d-Lys-Por (10 µM) (**D**) l-Lys-Por (10 µM) in PBS with 0.01% (shown in black) and 0.5% (shown in grey) of CA.

Circular dichroism (CD) is an optical technique that can confirm chiral interactions and has been used for characterizing porphyrin behavior [50]. Porphyrin signal detection in CD can imply the formation of aggregates originating from the porphyrin macrocycle [51,52]. The circular dichroic responses for Fmoc-d-Lys-Por and Fmoc-l-Lys-Por were recorded in the presence of 0.02% and 0.5% CA in PBS. Fmoc-d-Lys-Por showed a long wavelength positive band followed by a short wavelength negative band in the Soret band region in the presence of 0.02% CA (Figure 4). Fmoc-l-Lys-Por exhibited a long wavelength negative band followed by a short wavelength positive band in the Soret band region. On the other hand, Fmoc deprotected d-Lys-Por and l-Lys-Por showed a similar CD band, a long wavelength negative band, followed by a short wavelength positive band when 0.01% CA was used. Interestingly, the CD spectra peak intensity of all four complexes was greatly diminished in the presence of 0.5% CA in PBS. This implies full dissolution and prevention of aggregation with higher amounts of surfactant.

Generally, porphyrin-based photosensitizers can generate reactive oxygen species, specifically singlet oxygen, upon irradiation of appropriate wavelength light in the presence of oxygen [53,54]. To confirm singlet oxygen generation, d-Lys-Por and l-Lys-Por were incubated with singlet oxygen sensor green (SOSG), an indicator fluorescent dye that can detect singlet oxygen [55]. When the compounds were irradiated with a 405 nm light source, both complexes showed time-dependent generation of singlet oxygen (Figure S6). These results demonstrate the potential photosensitization of the synthesized complexes and potential for PDT. 

### 2.3. In Vitro Cell Studies

**Figure 5:**
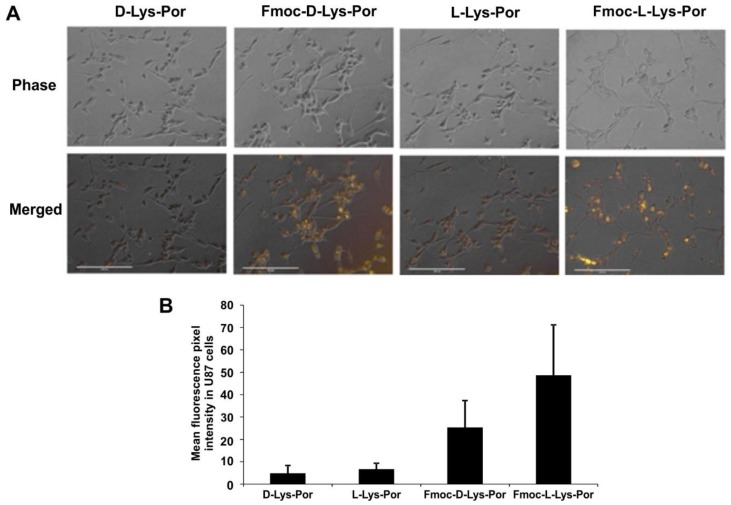
Cellular uptake of d-Lys-Por, Fmoc-d-Lys-Por, l-Lys-Por, and Fmoc-l-Lys-Por incubated with U87 cells at a concentration of 10 µM. (**A**) Phase image (top) and pyro (yellow) merged with phase (bottom) were captured using EVOS FL microscope. (**B**) Quantitative analysis of fluorescence brightness for *n* = 10 cells (mean +/− std. dev.). Fluorescence of the images was analyzed using ImageJ.

In order to assess uptake of the lysine–porphyrin conjugates in cancer cells, 10 µM of d-Lys-Por or l-Lys-Por (in the presence of 0.02% of CA) was incubated for 1 h with human glioblastoma U87 cells. Cells were examined for uptake by fluorescence microscopy. Interestingly, Fmoc-protected lysine complexes showed substantial uptake, whereas uptake of d-Lys-Por and l-Lys-Por was minimal (Figure 5A). Based on fluorescence microscope imaging analysis of single cells, cells incubated with Fmoc-d-Lys-Por had the greatest brightness, whereas l- and d-Lys-Por compounds had minimal uptake (Figure 5B). 

Because U87 cells are reported to express the LAT1 receptor [47,48,49], the lack of uptake of l-Lys-Por implies this targeting strategy was not effective. The lack of improved uptake of l-Lys-Por over the d-Lys-Por does not support specific uptake by the transporter, which prefers l-amino acids, although LAT1 has been reported to also recognize d-amino acids as well [56]. The improved uptake of Fmoc-protected compounds is not likely related to LAT1 since the Fmoc-protection would completely obscure recognition of the amino carboxy terminus. The uptake observed using d- or l-isomers was minimal and cells incubated with the different isomers had similar brightness. Most likely, the uptake of the Fmoc-protected lysine mTCPP relates to the higher hydrophobicity of those constructs, which could enable direct migration into cellular membranes. 

**Figure 6:**
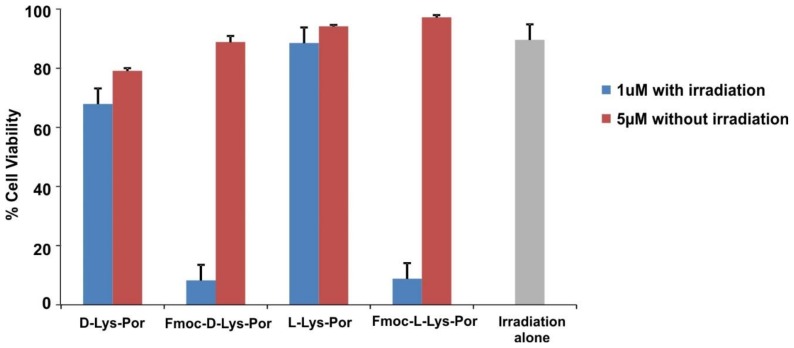
PDT-induced phototoxicity of U87 cells. d-Lys-Por, Fmoc-d-Lys-Por, l-Lys-Por, and Fmoc-l-Lys-Por were incubated with U87 cells for 12 h at indicated concentrations. Cells were then irradiated as indicated with a 405 nm laser diode at a fluence rate of 40 mW cm^−2^. Values show mean +/− std. dev. for *n* = 3 samples.

The PDT efficacy of d-Lys-Por and l-Lys-Por were investigated by incubating lysine-conjugated photosensitizers along with the Fmoc-protected complexes with U87 cells followed by irradiation with a blue laser diode (405 nm). Phototoxicity was determined by examining the toxicity induced by the complexes post-irradiation using an XTT assay. Cells were incubated with 1 µM of sample in cell media (in the presence of 0.02% CA) for 12 h. Following incubation, cells were washed with PBS and were incubated in media with FBS. Cells were then treated with blue light at a fluence rate of 40 mW cm^−2^. Cells treated with Fmoc-protected complexes induced significantly higher phototoxicity compared to d-Lys-Por and l-Lys-Por (Figure 6). The light dose response for Fmoc-l-Lys-Por phototoxicity is shown in Figure S7. Overall, the lack of phototoxicty of d-Lys-Por and l-Lys-Por is consistent with their lack of uptake into cells. The lack of uptake and lack of differential behavior between d- and l-isomers demonstrates that these compounds were not actively targeted via the LAT1 receptor. mTCPP complexes were able to induce cell death via PDT mechanisms when they were uptaken into cells and irradiated. None of the compounds exhibited substantial dark toxicity, even when they were incubated at a higher concentration of 5 µM. 

## 3. Conclusions

mTCPP was used as a platform for facile tetravalent conjugation of both d- and l-lysine, and Fmoc-protected d- and l-lysine. The resulting porphyrin conjugates could be dispersed in various surfactants including 1% cholic acid. The photophysical properties of d- and l-lysine mTCPP in aqueous solution were similar, with a prominent Soret band around 425 nm, minor Q bands from 520 to 650 nm, and fluorescence emission around 660 nm. Lysine-mTCPP was not uptaken by cells expected to express LAT1, but the Fmoc-protected complexes were. No difference between l- and d-isomer uptake was observed for either protected or deprotected compounds. These results do not support the premise that this particular lysine-mTCPP construct can target LAT1. The enhanced Fmoc-lysine-porphyrin compound cellular uptake was attributed to greater hydrophobicity of the compound. Once uptaken, mTCPP constructs could effectively induce phototoxicity for PDT applications. We conclude that mTCPP serves as a useful platform for assessing targeting ligands, owing to its commercial availability, ease of conjugation, and capabilities for imaging and photosensitization.

## 4. Materials and Methods

### Materials

Mesotetra(4-carboxyphenyl) porphyrin (mTCPP) was purchased from Frontier Scientific, Inc. (Logan, UT, USA), and *O*-(benzotriazol-1-yl)-*N*,*N*,*N*’,*N*’-tetramethyluronium hexafluorophosphate (HBTU) was purchased from Advanced Chem Tech Inc. (Louisville, KY, USA). Fmoc-Lys-OH and Fmoc-d-Lys-OH.HCl were purchased from Matrix Scientific and Anaspec Inc. (Fremont, CA, USA), respectively. *N*,*N*-Diisopropylethylamine (DIPEA) and 20% piperidine in DMF were purchased from Sigma (St. Louis, MI, USA). Pluronic F127 (Sigma), Cremophor EL (Sigma), Tween 80 (Sigma), sodium salt of Cholate (Sigma), methyl cellulose (Sigma), and Triton X (Sigma) were procured.

*General procedure for the synthesis of lysine-conjugated photosensitizers: Fmoc-d-Lys-Por and Fmoc-l-Lys-Por:* A 20 mL glass vial was charged with mTCPP (0.08 g, 0.1 mmol) and dissolved in 1 mL of anhydrous dimethylformamide (DMF), and the suspension was sonicated. To this suspension, a DMF solution of HBTU (0.152 g, 0.4 mmol) was added dropwise, followed by an addition of 5% DIPEA. The resulting solution was sonicated for 1 h at room temperature. Then, DMF solution containing Fmoc-d-Lys-OH.HCl (0.324 g, 0.8 mmol or Fmoc-Lys-OH (0.294, 0.8 mmol) was added dropwise to the above reaction mixture, and sonication was continued for another 45 min. The resulting mixture was neutralized with 10% citric acid and the crude compound was collected. The compound was purified using silica column with methylene chloride/methanol/30% NH_4_OH (70:15:2.5, *v*/*v*) as a mobile phase. After evaporating the solvent, a dark violet Fmoc-protected compound was obtained which was dried in vacuum. The obtained yields were 52% (0.1131 g) and 55% (0.12 g) for Fmoc-d-Lys-Por and Fmoc-l-Lys-Por, respectively. 

*NMR data:* Fmoc-d-Lys-Por: ^1^H-NMR (500 MHz, *d*^6^-DMSO): −δ −3.03 (br, s, 2H, NH-pyrrole), 1.18–1.83 (m, 24H, γLys, δLys, βLys), 3.29–3.75 (m, 8H, εLys), 3.86–4.19 (m, 4H, CH-Fmoc), 4.20–4.33 (m, 12H, CH_2_-Fmoc and αLys), 7.07 (s, 4H, –CH–NH–COO–, amide), 7.26–7.39 (M, 16H, Ar), 7.67–7.89 (m, 16H, Ar), 8.15–8.29 (m, 16H, Ar), 8.69 (s, 8H, Ar), 8.87 (s, 4H, –CO–NH–CH_2_–, amide). ^13^C-NMR (75 MHz, dmso): δ 174.10, 166.04, 155.63, 143.92, 143.75, 143.54, 143.48, 142.56, 140.58, 139.36, 137.38, 134.17, 133.93, 128.90, 127.47, 127.26, 126.96, 125.78, 125.19, 121.35, 119.98, 119.29, 109.74, 65.49, 55.01, 46.81, 31.45, 28.83, 22.94, 22.68. HR-MS (ESI): *m*/*z* 1096.4487 (calculated for [M + H]^2+^ 1096.4365). Fmoc-l-Lys-Por: ^1^H-NMR (500 MHz, *d*^6^-DMSO): −δ −3.02 (br, s, 2H, NH-pyrrole), 1.18–1.82 (m, 24H, γLys, δLys, βLys), 3.29–3.42 (m, 8H, εLys), 3.73–3.84 (m, 4H, CH-Fmoc), 4.19–4.34 (m, 12H, CH_2_-Fmoc and αLys), 7.01 (s, 4H, –CH–NH–COO–, amide), 7.26–7.29 (M, 16H, Ar), 7.67–7.86 (m, 16H, Ar), 8.17–8.43 (m, 16H, Ar), 8.70 (s, 8H, Ar), 8.87 (s, 4H, –CO–NH–CH_2_–, amide). ^13^C-NMR (75 MHz, dmso): δ 174.15, 166.05, 155.57, 143.94, 143.77, 143.56, 143.12, 142.53, 140.59, 139.34, 137.38, 134.22, 133.97, 128.89, 127.48, 127.26, 126.97, 125.77, 125.19, 121.31, 119.95, 119.30, 109.73, 65.40, 55.14, 46.71, 31.74, 29.07, 22.91, 22.53. HR-MS (ESI): *m*/*z* 1096.45251 (calculated for [M + H]^2+^ 1096.4365).

*Fmoc deprotection:* 20% piperidine in DMF (2.0 mL) was added to the Fmoc-protected compounds (Fmoc-d-Lys-Por and Fmoc-l-Lys-Por), and the mixture was vigorously stirred for 30 min. This procedure was repeated followed by washing with DMF (3 × 3.0 mL) and ether (3 × 1.0 mL). Deprotected compounds were purified by preparative TLC with isopropanol/NH_4_OH (8:1, *v*/*v*) as mobile phase. The obtained yields are 38% (0.030 g) and 44% (0.035 g) for d-Lys-Por and l-Lys-Por, respectively. d-Lys-Por: ^1^H-NMR (400 MHz, D_2_O at 80 °C): 1.50–1.94 (m, 24 H, γLys, δLys, βLys), 2.46 (m, 4H, αLys), 3.54–3.56 (m, 8H, εLys), and 7.24–7.99 (m, 24H, ArH, pyrrole). HR-MS (ESI): *m*/*z* 652.3030 (calculated for [M + H]^2+^ 652.3004). l-Lys-Por: ^1^H-NMR (400 MHz, D_2_O at 80 °C): −1.51–1.93 (m, 24 H, γLys, δLys, βLys), 2.49 (m, 4H, αLys), 3.54.3.60 (m, 8H, εLys), 7.18–8.02 (m, 24H, ArH, pyrrole). HR-MS (ESI): *m*/*z* 652.3028 (calculated for [M + H]^2+^ 652.3004).

*Physical and optical properties:* Absorbance was measured with a Lambda 35 UV/VIS or a Lambda XLS spectrophotometer (Perkin Elmer) using cuvettes with a 1 cm path length. Fluorescence intensity was measured with PTI instruments. ^1^H- and ^13^C-NMR spectrum were recorded with Varian Inova 300, 400, and 500 MHz instruments. The high resonance mass spectra (HR-MS) were recorded using Bruker Daltonics SolariX 12 Tesla Fourier Transform Ion Cyclotron Resonance Mass Spectrometer. Circular dichroic spectra were recorded using a Jasco PFD-350S/350L spectrometer.

*Singlet oxygen measurement:* A 50 µM stock solution of SOSG (Cat. # S36002, Life Technologies, Carlsbad, CA, USA) was prepared and 10 µL of the stock solution was added to 180 µL of PBS and 10 µL of mTCPP sample (Concentration: 5 µM). Fluorescence values of the samples were measured using TECAN at excitation/emission of 504/525 nm. Wells were irradiated with a blue laser diode at a fluence rate of 20 mW cm^−2^. Samples were read before and after irradiation of wells. Values obtained before irradiation were treated as reference and subtracted from post-treatment values. All measurements involving SOSG were done in triplicate and error bars indicate standard deviation from the mean.

*Fluorescence imaging, cell viability assay, and PDT studies:* Human glioblastoma (U87) cells were obtained from ATCC. Cells were cultured in Dulbecco’s modified Eagle’s medium with 10% fetal bovine serum (FBS) and 1% antibiotics. For cell viability and PDT, 1 × 10^4^ cells per well were seeded in a 96-well plate and were placed in a 37 °C environment for 24 h. Lysine-conjugated mTCPP samples dissolved in 0.02% CA were added to each well at 1 µM/5 µM/10 µM concentrations and incubated overnight. Fmoc-protected compounds were dissolved in 10 µL of methanol (final concentration: 0.01%) and diluted in 0.02% CA. Media-containing samples were removed, and cells were washed with PBS and incubated in fresh media containing FBS. Fluorescence microscopy was performed with an EVOS FL fluorescence microscope using a custom filter with 420 nm excitation and 670 nm emission. For microscopy studies, cells were incubated with 10 µM samples per well. Fluorescence images of single cells were analyzed using ImageJ. For PDT studies, cells incubated with samples were treated with a blue light source (405 nm) at a fluence rate of 40 mW cm^−2^. Post-treatment, cells were incubated for 24 h at 37 °C, and XTT assay was then performed. 

The XTT assay was performed by removing the media and washing the cells gently with 100 µL of PBS. Each well was loaded with 100 µL of PBS containing XTT (2,3-bis(2-methoxy-4-nitro-5-sulfophenyl)-2*H*-tetrazolium-5-carboxanilide) (50 µg/mL) and of PMS (*N*-methyl dibenzopyrazine methyl sulfate) (60 μg/mL) and incubated at 37 °C again. The 96-well plate was read at 450 nm and 630 nm (background) 2 h after incubation. (A_treated_ − A_blank_)/(A_untreated_ − A_blank_) × 100 was used to calculate cell viability values. All measurements were made in triplicate. 

## Supplementary Materials

Supporting information includes: S(1) ^1^H-NMR spectrum of Fmoc-d-Lys-Por and Fmoc-L-Lys-Por; S(2) ^13^C-NMR spectrum of Fmoc-d-Lys-Por and Fmoc-l-Lys-Por; S(3) HR-MS spectra of Fmoc-d-Lys-Por and Fmoc-l-Lys-Por; S(4) ^1^H-NMR spectrum of d-Lys-Por and l-Lys-Por in D_2_O at 80 °C and pH 10; S(5) HR-MS spectrum of d-Lys-Por and l-Lys-Por; S(6) Coincubation of SOSG and d-Lys-Por/l-Lys-Por for ROS measurement; S(7) PDT dose response of Fmoc-l-Lys-Por in vitro.
