# Supplementary material for: Assessing Photosensitizer Targeting Using Meso-Tetra(Carboxyphenyl) Porphyrin
Source: Molecules. 2018 Apr 12;23(4):892. doi: 10.3390/molecules23040892 (PMC6017280; doi:10.3390/molecules23040892)
Supplement: Supplementary file 1 [file molecules-23-00892-s001.pdf]

A.

Supplementary Materials

Assessing Photosensitizer Targeting using Meso-Tetra(carboxyphenyl) Porphyrin

Upendra Chitgupi[1], Jonathan F. Lovell[1]* and Venugopal Rajendiran[1,2]*

[1]Department of Chemistry, School of Basic and Applied Sciences, Central University of Tamil Nadu, Thiruvarur 610 005, India; rajendiran@cutn.ac.in
[2]Department of Biomedical Engineering, University at Buffalo, State University of New York, Buffalo, New York 14260, USA; jflovell@buffalo.edu

A.

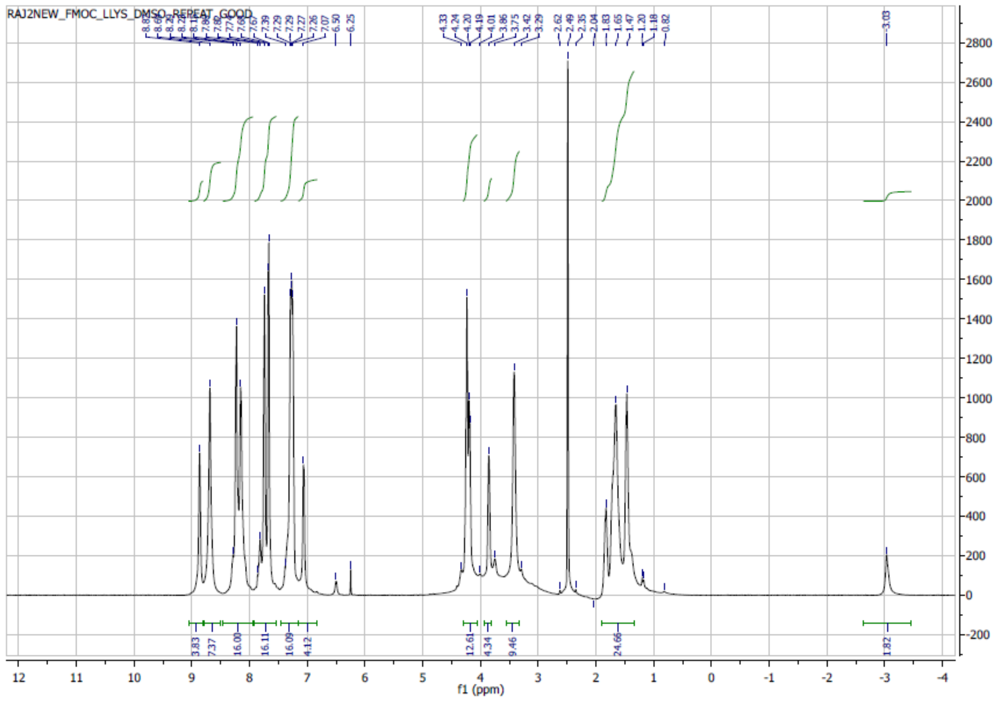

B.

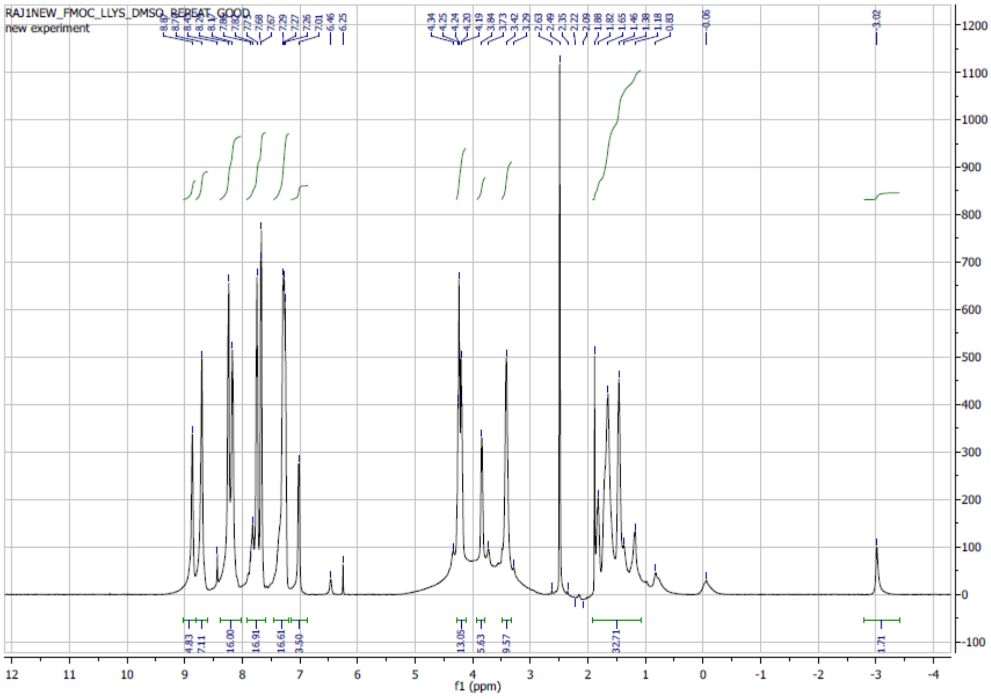

Figure S1. ^{1}H NMR spectrum of (A) Fmoc-D-Lys-Por and (B) Fmoc-L-Lys-Por in d^{6}-dmso 500 MHz Varian Inova Instrument.

A

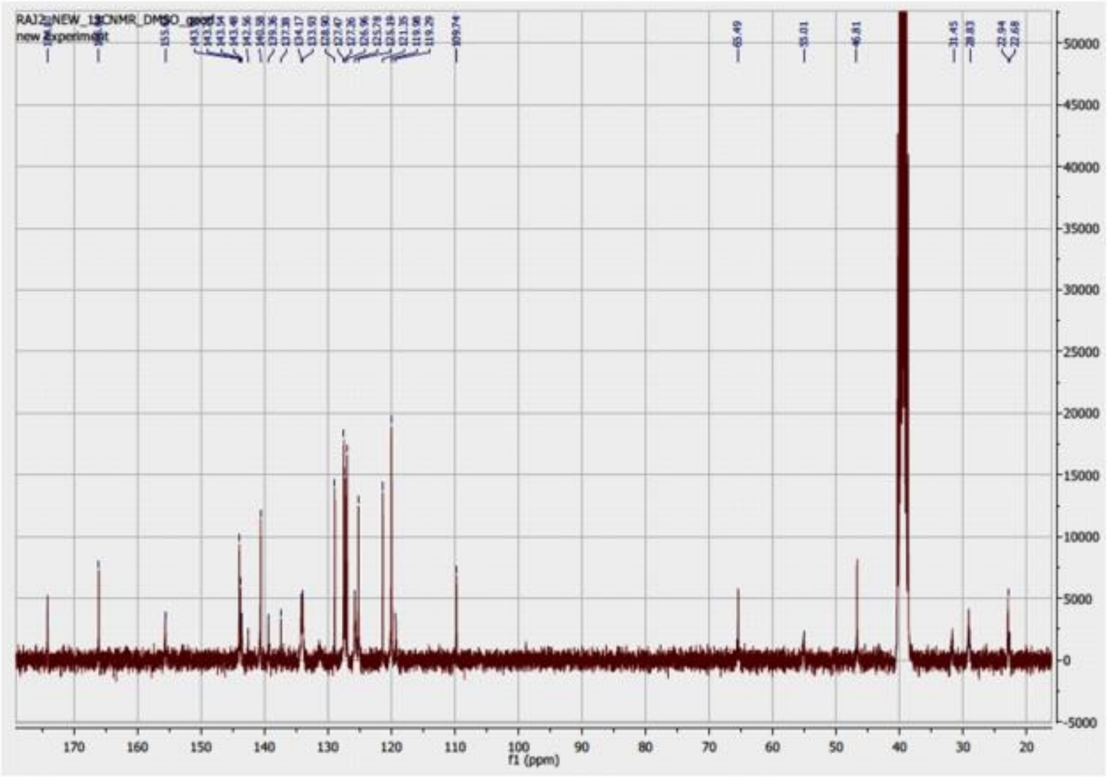

B

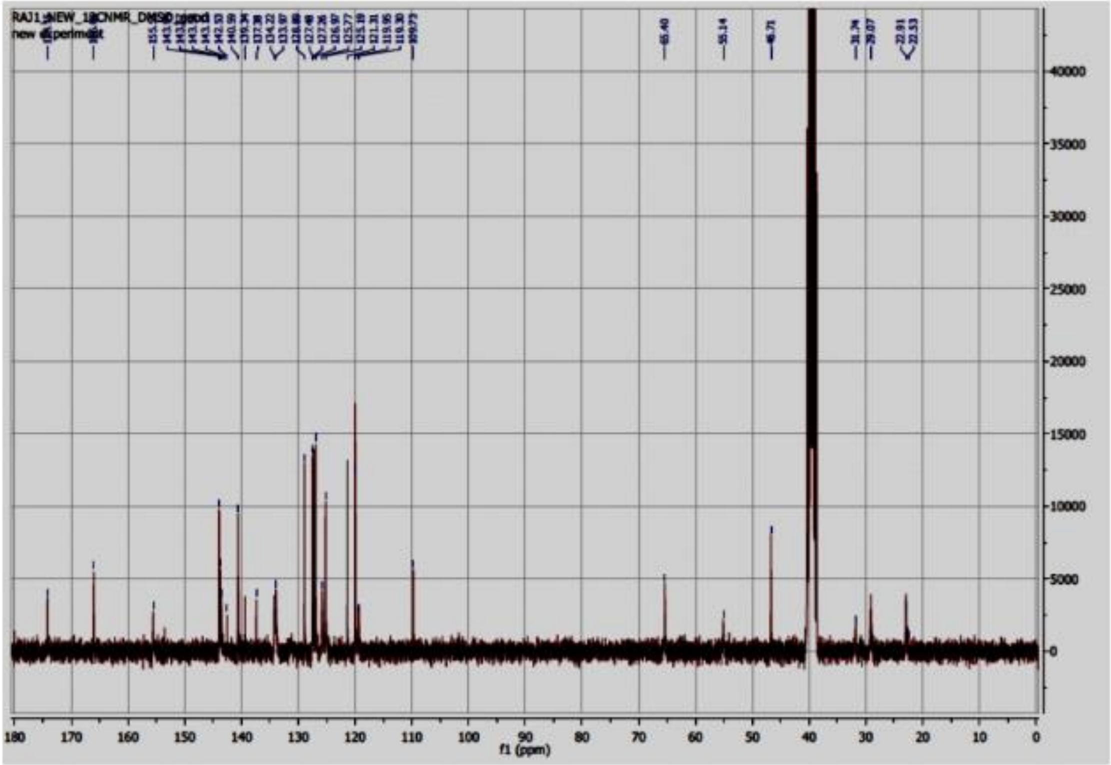

Figure S2. ^{13}C NMR spectrum of (A) Fmoc-D-Lys-Por (B) Fmoc-L-Lys-Por in d^6-dmso measured using 75 MHz Varian Inova Instrument.

A.

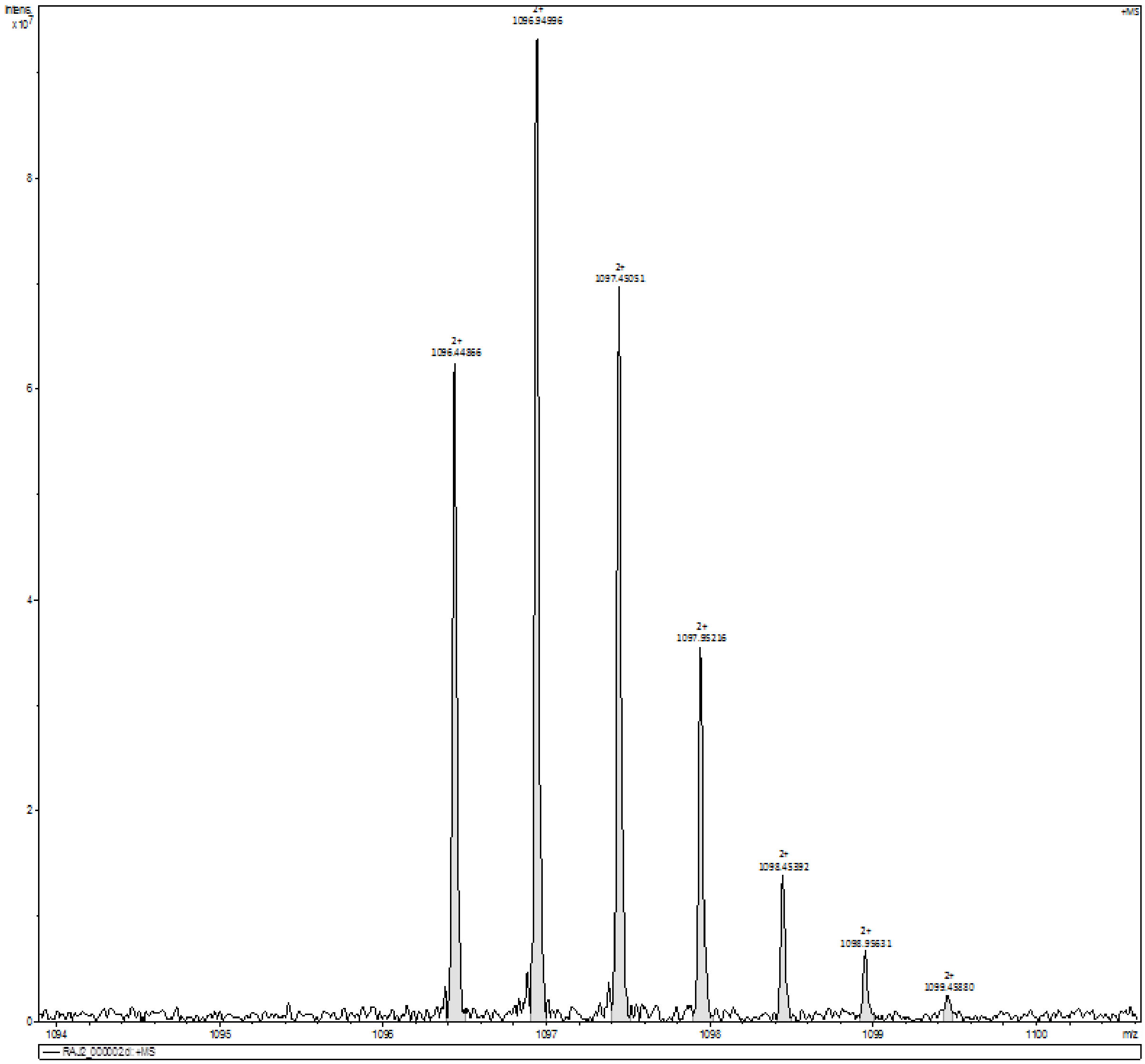

Intens.
x10^7
+MS
8
6
4
2
0
2+
1096.94996
2+
1097.45051
2+
1096.44866
2+
1097.95216
2+
1098.45382
2+
1098.95631
2+
1099.45880
1094
1095
1096
1097
1098
1099
1100
m/z
RAJ2_000002.d: +MS

B.

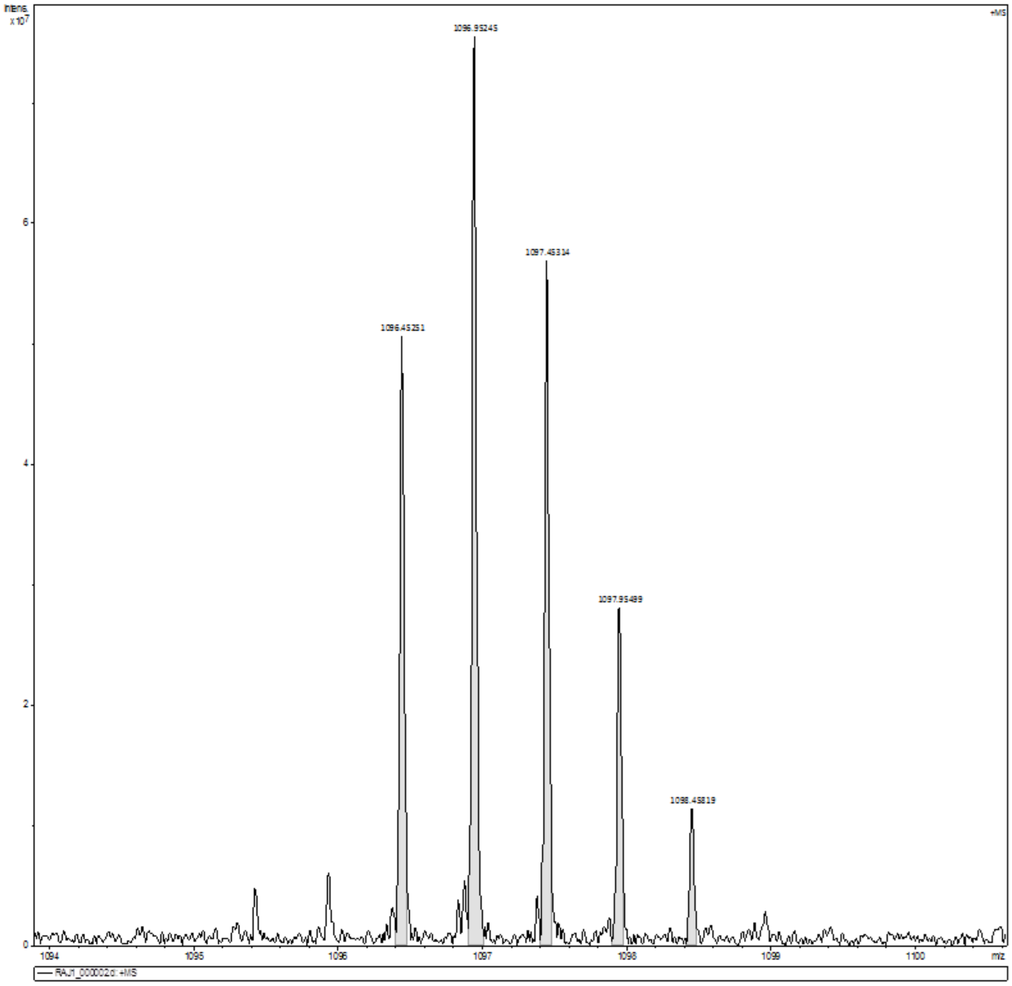

Figure S3. HR-MS spectrum of (A) Fmoc-D-Lys-Por and (B) Fmoc-L-Lys-Por.

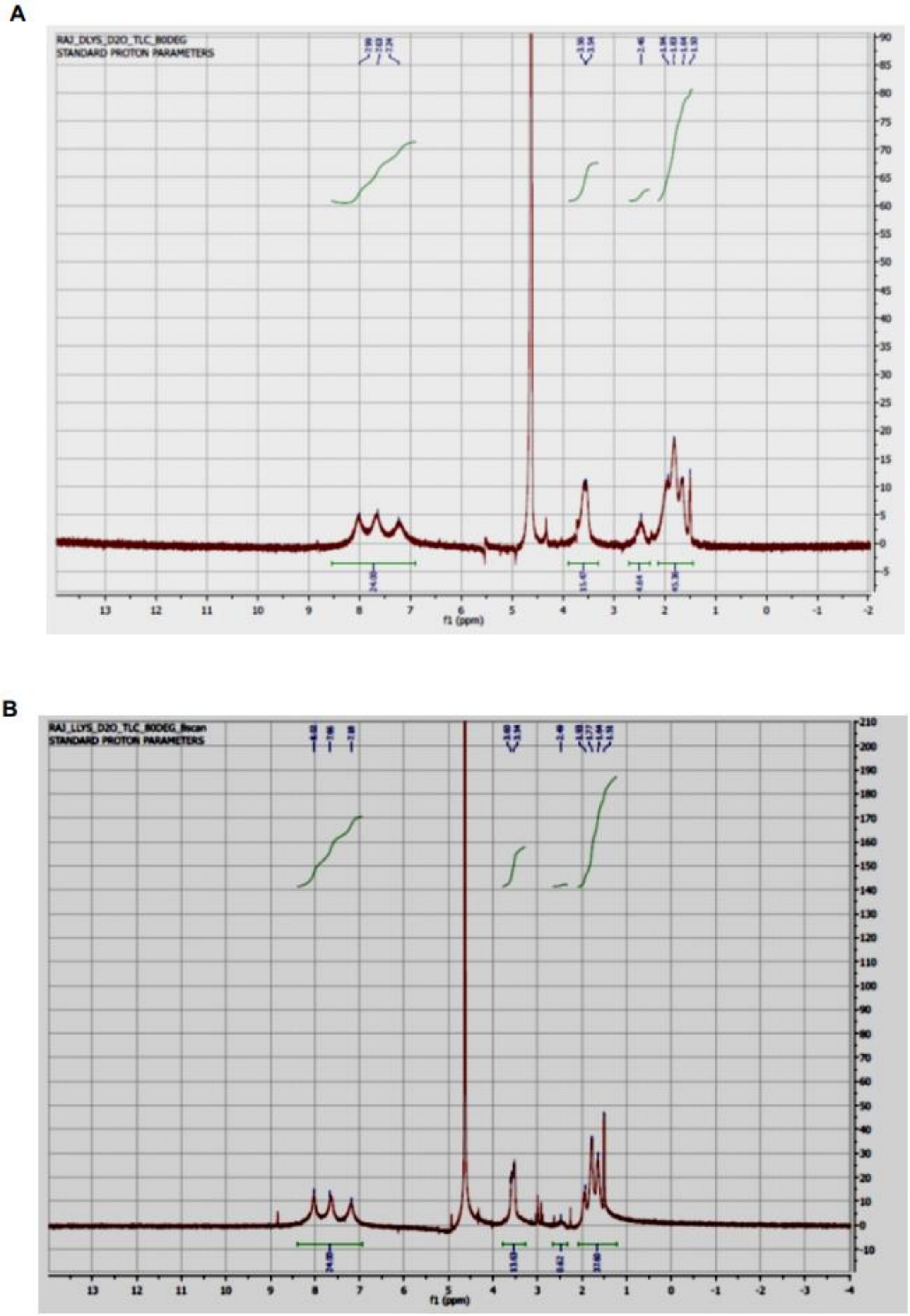

Figure S4. ^{1}H NMR spectrum of (A) D-Lys-Por (B) L-Lys-Por in D_2O at 80 °C pH 10.

A.

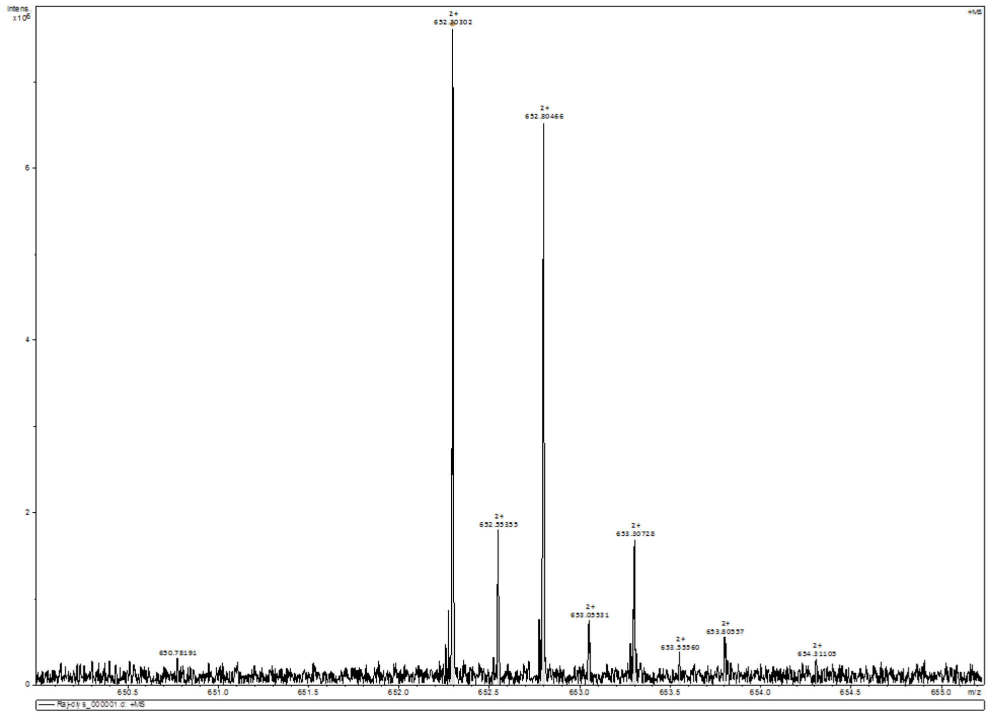

B.

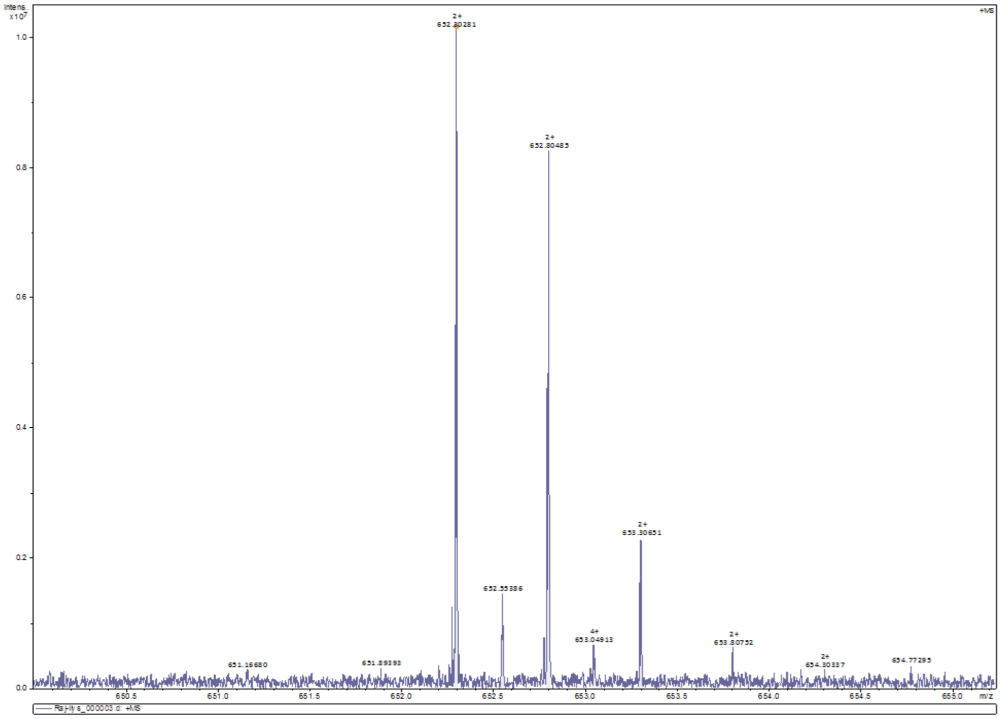

Figure S5. HR-MS spectrum of (A) D-Lys-Por and (B) L-Lys-Por.

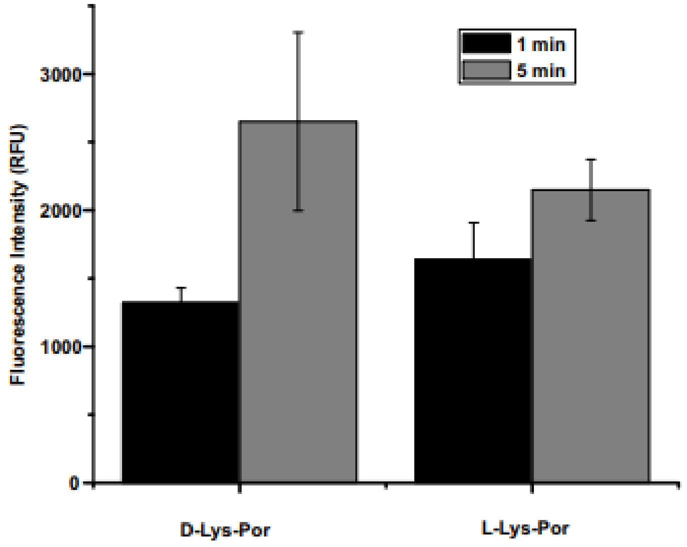

Figure S6. Coincubation of SOSG and D-Lys-Por/ L-Lys-Por for ROS measurement. Samples were incubated in media with FBS and irradiated with 405 nm laser. Fluorescence measurements were made with TECAN plate reader.

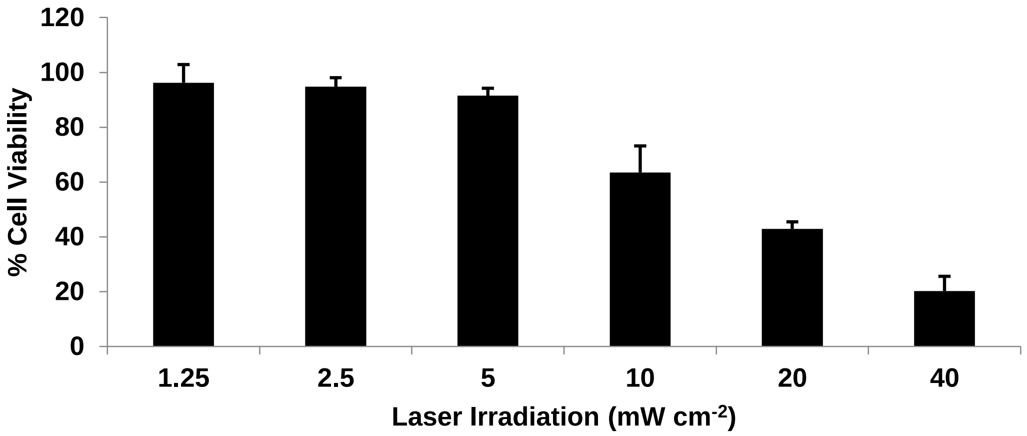

Figure S7. Light dose response (treated with a 405 nm laser diode) of Fmoc-L-Lys-Por following incubation with U87 cells. Data show mean +/- std. dev. for triplicate samples.
